# Developmental plasticity: Friend or foe?

**DOI:** 10.1093/emph/eox022

**Published:** 2018-02-05

**Authors:** Karin B Michels

**Affiliations:** Department of Epidemiology, Fielding School of Public Health, University of California, Los Angeles, CA, USA

**Keywords:** developmental plasticity, evolution, DOHaD, epigenetics

## Abstract

Developmental plasticity – the concept that adaptation to changing and unfavorable environmental conditions are possible but may come at the price of compromised health potentials – has evolutionary grounding as it facilitates survival but dissents with fundamental evolutionary principles in that it may advance the lesser fit. It is an important cornerstone of the Developmental Origins of Health and Disease (DOHaD). Unlike evolutionary adaptation developmental plasticity may be short-lived and restricted to one or few generations and inheritance is uncertain. Potential mechanisms include epigenetic modifications adopted in utero which may not transmit to the next generation; future insights may allow adjustments of the outcomes of developmental plasticity.

The duality of evolution and plasticity is intriguing and has evoked much debate [1]. The evolutionary grounding of plasticity is arduous to refute, as it permits the emergence of novel traits in response to selective environmental pressures. Nevertheless, rapidly altering nurture conditions do not seamlessly integrate with evolutionary selection across multiple generations. Moreover, developmental plasticity in particular often contradicts the evolutionary principle of ‘selection of the fittest’. Whether following a developmental constraints or a predictive response model, which Lee *et al.* [2] demarcate in their article in this issue of the journal, developmental plasticity allows nurture of an inferior variant by permitting survival in an adverse environment (under the constraints model) and/or sanctions a high-risk strategy of programming the organism to early conditions anticipating a steady state in the future while enduring a considerable chance of mismatch (under the predictive response model). Effective evolution would not tolerate emergence of such compromised variants with poor prospects but likely select for variants with superior traits.

The concept of developmental plasticity has been embraced by the followers of the Barker-hypothesis (subsequently termed Developmental Origins of Health and Disease [DOHaD]) as a suitable model for epidemiologic observations linking the intrauterine environment to later life health [3]. This concept delineates the ability of the fetus to adapt to the intrauterine environment at the cost of modifying long-term health prospects. Most DOHaD phenomena link a resource-deprived or -compromised environment, characterized by maternal starvation, stress or disease, or fetal hormonal or chemical imbalance to chronic disease outcomes decades later. This thrifty phenotype [4] is largely viewed as the inability to properly adjust to environmental perturbations that lie beyond any anticipated variation. A redirected program releases the fetus into an uncertain future.

Plasticity of an organism is assumed greatest at conception and to dwindle thereafter [5]. With increasing age plasticity is compromised. Risk of disease generally escalates with age and declining plasticity. Nevertheless, plasticity can be evoked throughout the lifecourse by perturbed environments such as seasonal famine diminishing fertility in sub-Saharan populations [6]. Interestingly, susceptibility to disease varies throughout lifetime with vulnerable windows tied to important developmental milestones, such as conception, birth, puberty, pregnancy and menopause; however, the intrauterine interval represents an special opportunity for heightened disease propensity for the vulnerable fetus and coincides with the developmental plasticity concept of reaching beyond the boundaries of anticipated programing in response to an unusual environment.

While genetic traits favored by natural selection slowly evolve over large time-intervals—reacting to environmental variations—developmental plasticity or the thrifty phenotype allows short-term responses to the environment without genome alterations. The mechanisms allowing such immediate defense are poorly understood and a current focus of DOHaD research. The concept of plasticity–phenotypic accommodation in the absence of genetic change-aligns with epigenetic principles: the propensity of phenotypic modification without genetic change. Indeed, epigenetic mechanisms represent a likely explanation for much of the DOHaD phenomena. Most of the epigenetic code is determined *in utero*, and the intrauterine environment will imprint its marks on the epigenetic signature. The stability of such marks throughout the lifecourse remains unclear, and potential reversibility would allow corrections of plasticity-misdirected prospects. Although DNA methylation, chromatic structure, histone modification and miRNA activity are malleable, the complexity of this delicate and intertwined epigenetic architecture has not been disassembled. What has emerged, however, is that all components are impressionable by the environment and in particular DNA methylation may offer stable, long-lasting profiles that may affect disease propensity [7]. 

Is developmental plasticity a survival plan gone awry? Evolutionary interests should not support survival of the unfit. The concepts, however, may be less divergent than apparent. Evolution fosters multi-generational adaptation to the ever-changing environment to secure long-term survival of the genetic lineage, whereas developmental plasticity allows short-term tolerance possibly to avoid instant extinction within the same generation—at the cost of inferior health. A big unknown is whether passing the newly acquired traits and ‘quick fix’ to the next generation is part of that master plan. Do they offer a survival advantage to the offspring? If epigenetics is indeed the underlying mechanistic tool inheritance may not be the intention. A double-layer of epigenetic erasure, two distinct demethylation cycles—one prior to and one subsequent to conception—aims to ensure the deterrence of passing any methylation marks to the next generation [8]. Transgenerational epigenetic inheritance would require incomplete erasure. While this has been the focus of much debate, little confirmatory evidence exists in humans, although some other species display an epigenetic memory [9]. In the context of DOHaD, not conserving these hastily adopted programs might be in the best interest of the evolutionary goal to optimize fitness.

With much exploration ongoing in this field, our cursory understanding of these complex but fundamental processes will be much enriched and may stipulate unknown prospects for prevention.

**Conflict of interest:** None declared.
